# Coagulase-Negative Staphylococci Clones Are Widely Distributed in the Hospital and Community

**DOI:** 10.3390/pathogens10070792

**Published:** 2021-06-23

**Authors:** Luiza Pinheiro-Hubinger, Danilo Flávio Moraes Riboli, Lígia Maria Abraão, Eliane Patricia Lino Pereira Franchi, Maria de Lourdes Ribeiro de Souza da Cunha

**Affiliations:** 1 Department of Chemical and Biological Sciences, Microbiology and Immunology Sector, Institute of Biosciences of Botucatu, Universidade Estadual Paulista-UNESP, Botucatu 18618-970, Brazil; l.pinheiro@unesp.br (L.P.-H.); danilo.riboli@ibb.unesp.br (D.F.M.R.); ligia.abraao@ymail.com (L.M.A.); fliane24@yahoo.com.br (E.P.L.P.F.); 2 Department of Anatomic Pathology, Instituto Lauro de Souza Lima, Bauru 17034-971, Brazil

**Keywords:** coagulase-negative staphylococci, SCC*mec*, PFGE, MLST

## Abstract

Coagulase-negative staphylococci (CoNS) may be considered contaminants when isolated from clinical specimens but may also be a cause of true infection. This study aimed to compare the clonality and SCC*mec* type of a collection of CoNS isolated from blood cultures of inpatients, nasal swabs of healthy individuals, and patients with chronic wounds, all from the same community, using SCC*mec* typing, pulsed-field gel electrophoresis (PFGE), and MLST. *Staphylococcus epidermidis*, exhibited high clonal diversity, but hospital and community clusters were observed. Nosocomial *S. epidermidis* clones belonged to sequence types ST2, ST6, and ST23. Some *Staphylococcus haemolyticus* clones were found to circulate in the hospital and community, while *Staphylococcus saprophyticus* exhibited very high clonal diversity. *Staphylococcus lugdunensis*, *Staphylococcus warneri*, and *Staphylococcus capitis* revealed several isolates belonging to the same clone in the hospital and community. The detection of different SCC*mec* types within the same cluster indicated high diversity. *S. epidermidis* was associated with SCC*mec* I and III, *S. haemolyticus* with I and II, *S. capitis* with type V, *Staphylococcus hominis* with *mec* complex type A and *ccr1*, and *S. warneri* and *S. saprophyticus* with SCC*mec* I. The generation of elements and new combinations of cassette genes were highly associated with CoNS isolates, suggesting that SCC*mec* may not be a good marker of clonality in these bacteria.

## 1. Introduction

Coagulase-Negative Staphylococci (CoNS) are colonizers of human and animal skin and mucosa [1,2,3]. The group of CoNS is composed of several species, including *Staphylococcus epidermidis*, *Staphylococcus haemolyticus*, *Staphylococcus saprophyticus*, *Staphylococcus capitis*, *Staphylococcus warneri*, *Staphylococcus hominis*, *Staphylococcus lugdunensis*, *Staphylococcus simulans*, *Staphylococcus caprae*, *Staphylococcus cohnii*, *Staphylococcus xylosus*, and *Staphylococcus schleiferi* [4]. Although they live in symbiosis with their host, CoNS can cause important infections in case of breakdown of the skin barrier [4]. Therefore, CoNS are considered essentially opportunistic agents that use organic situations to produce serious infections [3,5]. The pathogenic potential of CoNS is mainly related to some virulence factors, especially biofilm, but also toxins and antimicrobial resistance. Some criteria are used to determine if a CoNS is a true cause of infection, such as absence of infection caused by another microorganism, presence of two or more isolates of the same species, improvement of symptoms after therapy, presence of foreign body, time for positivity less than 16 hours, and antibiotic resistance [6,7]. It is a known fact that CoNS infections may be seriously impacted by antimicrobial resistance.

Antimicrobial resistance is a global challenge for the treatment of infections caused by staphylococci and other bacteria, especially hospital-acquired infections. The estimated prevalence of methicillin resistance is 20% among community isolates [8] and more than 80% among hospital isolates [9]. This resistance is usually mediated by a modified penicillin-binding protein 2 (PBP2), which is encoded by the *mecA* gene. The gene is carried by a mobile genetic element, the staphylococcal chromosome cassette *mec* (SCC*mec*), which is considered a vehicle for the exchange of resistance factors between CoNS and *S. aureus* [10]. In addition, SCC*mec* is used to characterize the phylogenetic relationship among *Staphylococcus* isolates.

The molecular typing of hospital *S. epidermidis* isolates using different tools has shown considerable diversity within this species [11]. Pulsed-field gel electrophoresis (PFGE) is widely used in studies investigating the local clonality of bacterial strains and is efficient in identifying staphylococcal profiles. Studies using Multilocus Sequence Typing (MLST) have shown that the population structure of *S. epidermidis* in hospitals is composed of a main lineage with high genetic diversity, clonal complex 2 (CC2) [12,13,14]. The combination of SCC*mec* and PFGE profiling has provided short- and long-term epidemiological data that are consistent with the results of MLST [14]. However, little is known about the clonal diversity of non-epidermidis CoNS species or about colonization CoNS isolates and their clonal relationship with isolates from infection sites.

Therefore, this study aimed to evaluate the clonal profile and SCCmec type of S. epidermidis, S. haemolyticus, S. hominis, S. warneri, S. capitis, S. lugdunensis, S. saprophyticus, Staphylococcus pasteuri, S. simulans, S. xylosus, and S. cohnii isolated from blood cultures of hospital inpatients, nasal swabs of healthy individuals, and patients with chronic wounds attending basic health units, all from the same community.

## 2. Results

The following species were isolated from nasal swabs: 57 (39.3%) *S. epidermidis*, 18 (11.7%) *S. haemolyticus*, 27 (17.5%) *S. warneri*, 19 (12.3%) *S. lugdunensis*, 5 (3.2%) *S. capitis*, 12 (7.8%) *S. saprophyticus*, 2 (1.3%) *S. simulans*, 3 (2%) *S. pasteuri*, 1 (0.6%) *S.* *xylosus,* and 1 (0.6%) *S. cohnii*.

Among the wound isolates, we identified 3 (13.6%) *S. epidermidis*, 7 (31.8%) *S. haemolyticus*, 5 (22.7%) *S. warneri*, 1 (4.5%) *S. hominis*, 1 (4.5%) *S. lugdunensis*, 1 (4.5%) *S. simulans*, 2 (9%) *S. capitis*, and 2 (9%) *S. saprophyticus*. The blood culture isolates included 40 (30.3%) *S. epidermidis*, 28 (21.2%) *S. haemolyticus*, 7 (31.8%) *S. warneri*, 22 (16.7%) *S. hominis*, 10 (7.6%) *S. lugdunensis*, and 25 (19%) *S. capitis*.

**Figure 1:**
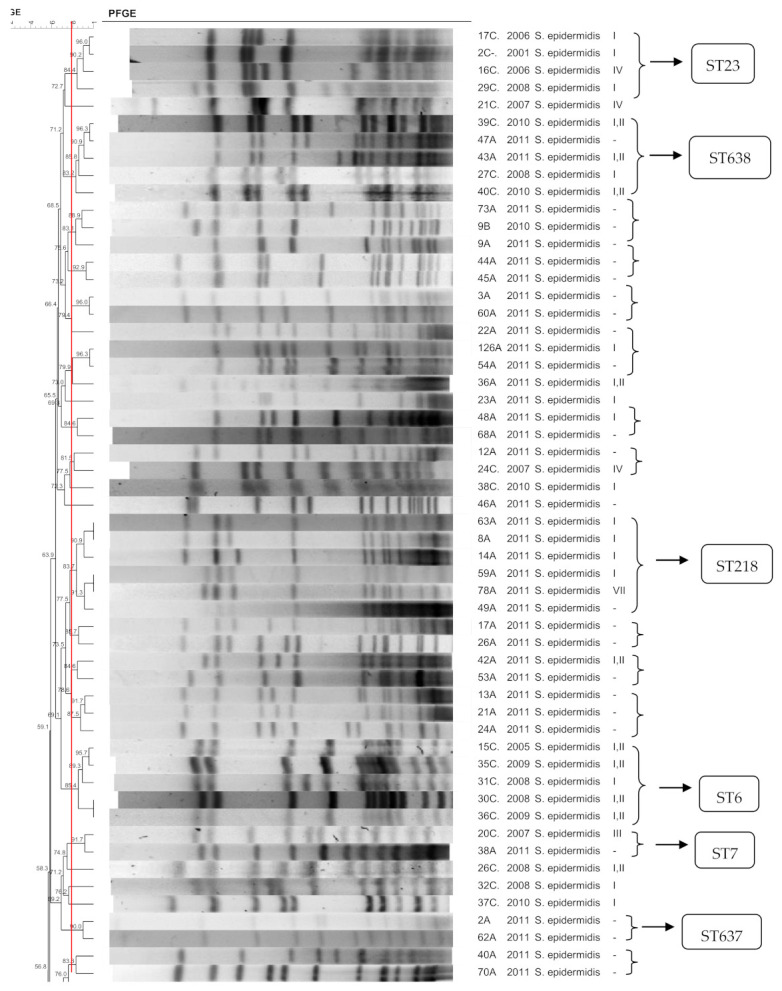

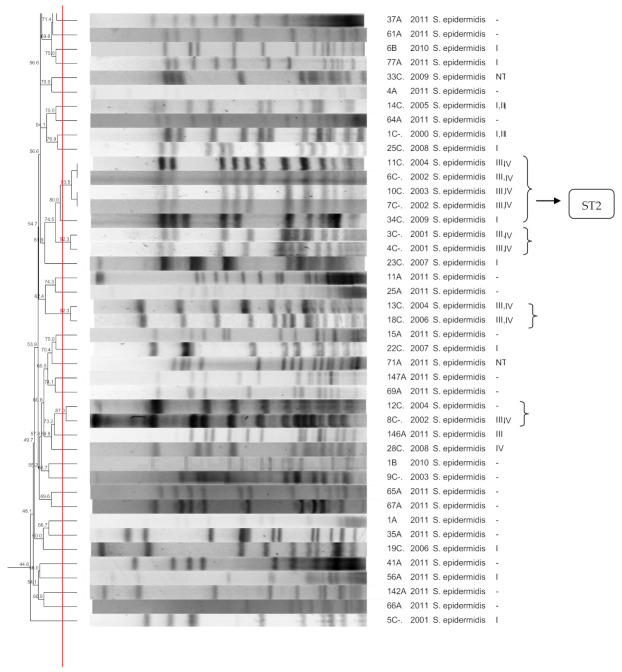
Dendrogram generated by Dice/UPGMA analysis (*Bionumerics Applied Maths*), from PFGE-*SmaI* profiles of *S. epidermidis* isolated from nasal swabs (**A**), wounds (**B**), and blood cultures (**C**). Square brackets highlight the clusters (>80% similarity). Roman numerals represent the SCC*mec* type.

**Figure 2:**
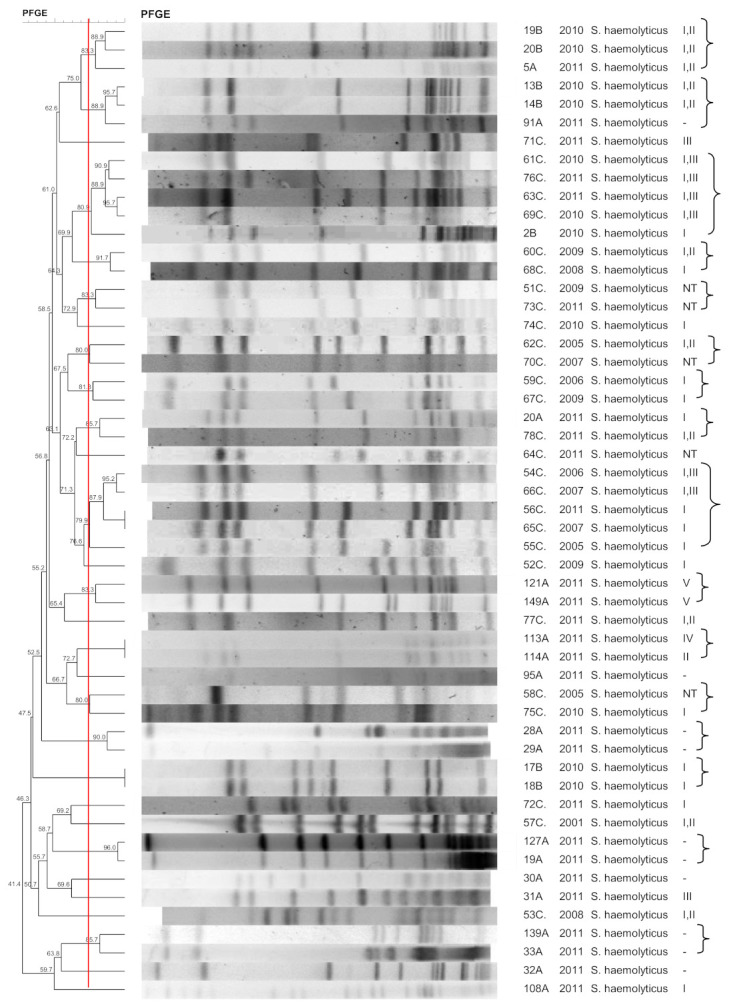
Dendrogram generated by Dice/UPGMA analysis (Bionumerics Applied Maths), from PFGE-*SmaI* profiles of *S. haemolyticus* isolated from nasal swabs (**A**), wounds (**B**), and blood cultures (**C**). Square brackets highlight the clusters (>80% similarity). Roman numerals represent the SCC*mec* type.

**Figure 3:**
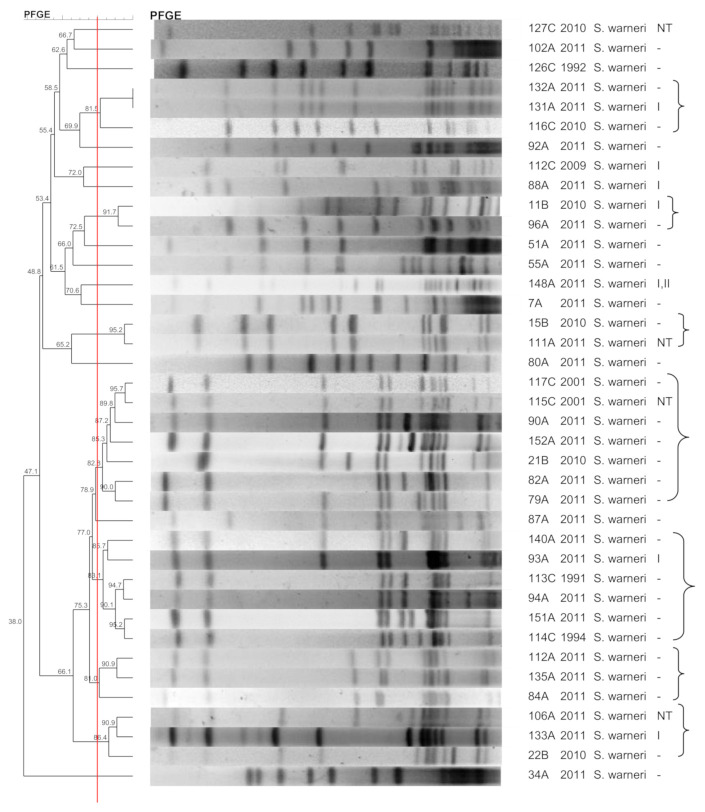
Dendrogram generated by Dice/UPGMA analysis (*Bionumerics Applied Maths*), from PFGE-*SmaI* profiles of *S. warneri* isolated from nasal swabs (**A**), wounds (**B**), and blood cultures (**C**). Square brackets highlight the clusters (>80% similarity). Roman numerals represent the SCC*mec* type.

**Figure 4:**
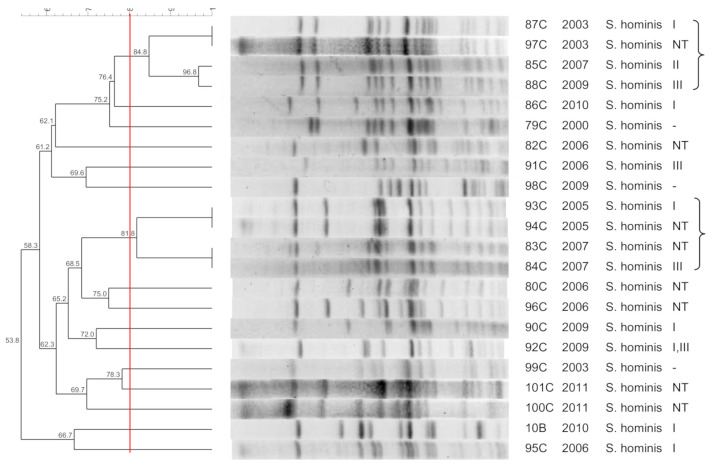
Dendrogram generated by Dice/UPGMA analysis (*Bionumerics Applied Maths*), from PFGE-*XhoI* profiles of *S. hominis* isolated from wounds (**B**), and blood cultures (**C**). Square brackets highlight the clusters (>80% similarity).

**Figure 5:**
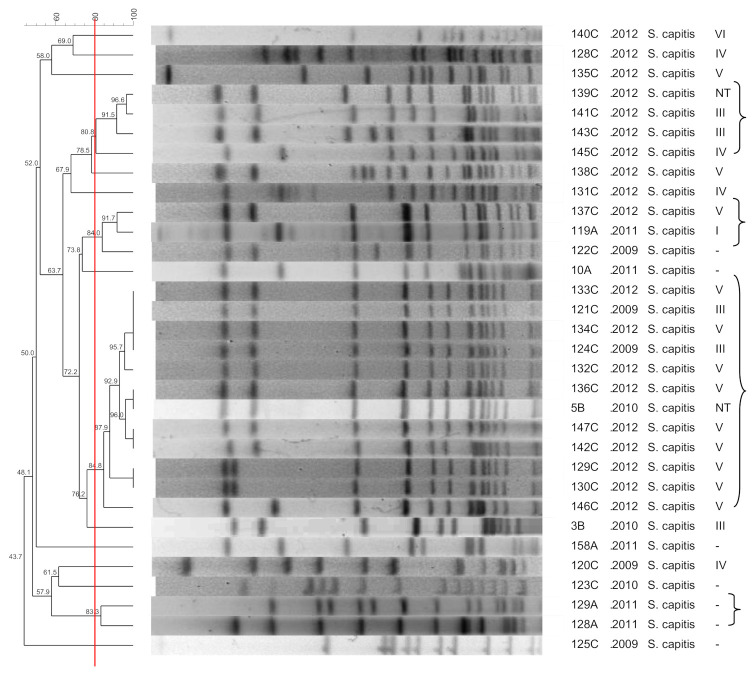
Dendrogram generated by Dice/UPGMA analysis (*Bionumerics Applied Maths*), from PFGE-*SmaI* profiles of *S. capitis* isolated from nasal swabs (**A**), wounds (**B**), and blood cultures (**C**). Square brackets highlight the clusters (>80% similarity). Roman numerals represent the SCC*mec* type.

**Figure 6:**
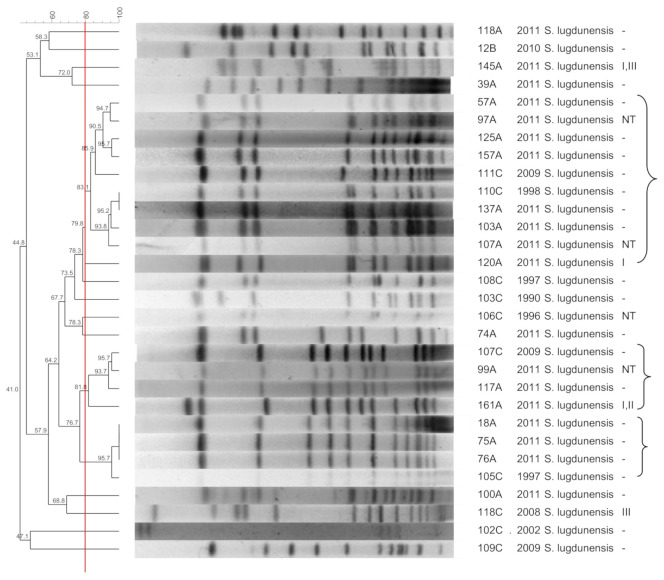
Dendrogram generated by Dice/UPGMA analysis (*Bionumerics Applied Maths*), from PFGE-*SmaI* profiles of *S. lugdunensis* isolated from nasal swabs (**A**), wounds (**B**), and blood cultures (**C**). Square brackets highlight the clusters (>80% similarity). Roman numerals represent the SCC*mec* type.

**Figure 7:**
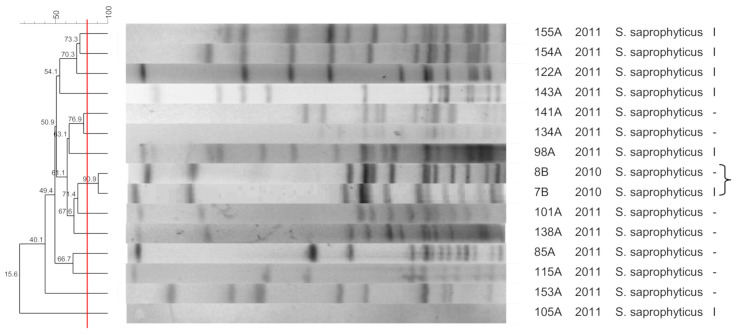
Dendrogram generated by Dice/UPGMA analysis (*Bionumerics Applied Maths*), from PFGE-*SmaI* profiles of *S. saprophyticus* isolated from nasal swabs (**A**) and wounds (**B**). Square brackets highlight the clusters (>80% similarity). Roman numerals represent the SCC*mec* type.

**Figure 8:**
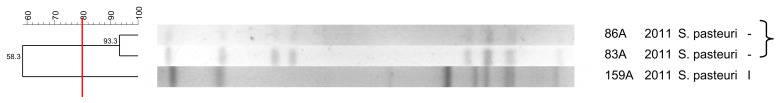
Dendrogram generated by Dice/UPGMA analysis (*Bionumerics Applied Maths*), from PFGE-*SmaI* profiles of *S. pasteuri* isolated from nasal swabs (**A**). Square brackets highlight the clusters (>80% similarity). Roman numerals represent the SCC*mec* type.

Figure 1, Figure 2, Figure 3, Figure 4, Figure 5, Figure 6, Figure 7 and Figure 8 show the dendrograms of PFGE types for *S. epidermidis*, *S. haemolyticus*, *S. warneri*, *S. hominis*, *S. lugdunensis*, *S. capitis*, *S. saprophyticus*, and *S. pasteuri*, respectively. The similarity of the three *S. simulans* strains was less than 60% (threshold was >80%), and these isolates are therefore not represented in a dendrogram.

A total of 185 (62%) isolates were positive for the *mecA* gene, including 48 from the community collection (33%), 21 from wounds (100%), and 116 from blood cultures (88%). Table 1 shows the number and percentage of SCC*mec* types according to sampling site. The results for the non-typable isolates (cases in which only the internal control for *mecA* was amplified in Protocol 2 are not shown) are presented in Table 2.

Some clones were chosen for the MLST analyses, including (i) the largest clones containing the largest number of strains, (ii) a cluster formed by a colonization isolate and a clinical isolate, and (iii) a colonization clone. The sequence types are presented in Figure 1. Two strains (2A and 47A) exhibited a new profile and were named sequence types ST637 and ST638, respectively. Table 3 shows the diversity indexes based on the clonal profiles. The higher the diversity index, the less similar are the strains from that specific group.

## 3. Discussion

This study aimed to compare clinical, commensal, and wound CoNS strains in order to evaluate the clonal connection of isolates belonging to different species and of different origins. This type of study is the first step to understand the diversity of a species collection and to determine the linkage between subpopulations of strains, especially between colonization and infection isolates, or between susceptible and resistant strains [15].

Due to being isolated from several clinical specimens, CoNS may be wrongly described as contaminants, despite being the true cause of infection in many cases. Diverse clinical and microbiological factors are used to classify CoNS as true causes of infection or as contaminants [16]. Some studies show high antimicrobial resistance rates as factors associated with CoNS causing infection [16].

### 3.1. S. epidermidis

The *S. epidermidis* isolates comprised a highly diverse collection of strains, both in the hospital and in the community, as reported previously [17,18,19,20], despite the presence of three hospital clusters, some clones in the community, and one cluster including blood culture and nasal isolates. Isolates of the last cluster were typed by MLST and belonged to a new ST638, closely related to ST151 and ST228. This finding suggests evolutive advantages of this clone, allowing its circulation between the hospital and community [12].

Clones composed of hospital isolates belonged to ST2, ST6, and ST23. ST23 has been associated with linezolid resistance, carrying mutations in the 23S rRNA gene as well as the *cfr* plasmid [21,22,23,24,25]. ST2 has been described in clinical and colonization isolates [12,21,26] but is more frequently found in hospital strains [16]. ST6 is often observed in clinical and colonization isolates [11] and is one of the main types among hospital isolates [13]. In the present study, ST6 was associated with SCC*mec* I and III, in contrast to a previous study in which ST6 was associated with SCC*mec* II and V [13], as well as with linezolid resistance [27].

One strain clustered to a hospital–community clone belonged to ST7, a sequence type that has been associated with SCC*mec* IV [21], close to the hospital–community interface. As in the present work, susceptibility of ST7 to methicillin has been reported [16]. Two community clones were identified as ST218 and ST637. In a previous study, ST218 was described in *S. epidermidis* causing bloodstream infection [28].

### 3.2. S. haemolyticus

The *S. haemolyticus* isolates exhibited some clonal diversity, forming small clusters. One cluster contained seven hospital strains, another contained two commensal and two wound strains, and one cluster contained one blood culture and one wound isolate. The chronic wound isolates were genetically related to nosocomial and commensal strains, highlighting their opportunistic role. Indeed, the high plasticity of *S. haemolyticus* was demonstrated by genome sequencing, which revealed a high frequency of insertion sequences and transposons [29]. The high clonal diversity of *S. haemolyticus* has also been described in previous studies [20,30]. Occasionally, certain *S. haemolyticus* clones may predominate over others in the hospital [31]. Their genomic plasticity seems to contribute to the heterogeneity of the species, and small clones may appear at close locations within the hospital or community, but circulation within and between these environments does not seem to be common.

### 3.3. S. hominis

*S. hominis* was the only CoNS isolate that was not properly restricted by *Sma*I. Bouchami et al. [32] observed the same phenomenon, as well as low clonality of this species. Our results show high diversity of *S. hominis* within the hospital, despite the presence of a clone circulating for 7 years and another for 3 years. However, most strains seem to originate from the patient’s microbiota. Comparison with *S. hominis* isolated from healthy individuals outside the hospital would permit a better understanding of the diversity of this species. The absence of *S. hominis* in nasal swabs is a limitation of the study.

A high prevalence of SCC*mec* III in *S. hominis* has been reported in Brazil [33]. In the present study, 20% of isolates with non-typed SCC*mec* carried *mec* complex type A and *ccr1*, as previously reported [32,34]. The high frequency of the *mec*A complex suggests that *S. hominis* may be a reservoir of this element.

### 3.4. S. saprophyticus

Nasal isolates of *S. saprophyticus* did not form clusters. The two *S. saprophyticus* strains isolated from the wounds of two patients exhibited a similar profile. On the other hand, Sousa et al. [35] showed high clonality of *S. saprophyticus* strains causing community-acquired urinary tract infections. Widerstrom et al. [36] found a very heterogeneous population consisting of several *S. saprophyticus* clones associated with urinary tract infection and the lack of a specific uropathogenic clone; however, there were clusters of highly related isolates. These data demonstrate a high diversity of this species among commensal isolates, with a low probability of causing infections. Certain genotypes may have biological advantages that facilitate their persistence as causes of infection. The same genotype found in different patients suggests a common source of contamination with this microorganism. However, it remains unclear which intrinsic bacterial factors facilitate infection.

### 3.5. S. lugdunensis

*S. lugdunensis* is less commonly isolated than other CoNS. When truly causing infection, the presence of *S. lugdunensis* is important since the course of infection is similar to that of *S. aureus* [37]. *Staphylococcus lugdunensis* is associated with increased symptomatic manifestation and a shorter interval between surgery and infection when compared to *S. epidermidis* and *S. aureus* [38]. Some authors suggest that any *S. lugdunensis* isolate should be considered a pathogen until proven otherwise [37].

The *S. lugdunensis* isolates formed two main clusters that contained commensal and clinical strains, with high similarity. The clustering of strains isolated within a period of up to 15 years suggests a low level of genetic diversity within this species, as shown previously [15,39]. The genetic structure of this population also implies that most commensal strains have the ability to cause infection, at least locally, or that hospital isolates may be spreading to the community, reaching the healthy population. Furthermore, the high similarity among the restriction patterns may also indicate that this is due to the intrinsic homogeneity of *S. lugdunensis* rather than to an epidemiological link among the isolates [15]. 

### 3.6. S. warneri

Although *S. warneri* is rarely isolated from human infections, this species was relatively common in the nasal and wound samples and less frequent in blood cultures. Species diversity was low, with some clusters including wound, nasal, and blood culture isolates. The observation of a PFGE type with several representatives demonstrates the circulation of a single clone in the hospital and community. Previous studies have shown a higher diversity of *S. warneri* [40] and the dissemination of a single clone among health professionals [41]. The paucity of molecular epidemiology studies involving *S. warneri* hinders understanding the clonality of this species. To our knowledge, there are no genetic studies involving a large collection of *S. warneri* strains, and our results may therefore contribute to a better understanding of *S. warneri* dissemination.

### 3.7. S. capitis

The heterogeneity of *S. capitis* was low among blood culture isolates. De Silva et al. [42], studying sepsis, blood culture contaminants, and colonization strains, found that all 29 *S. capitis* isolates belonged to one clone. However, the authors only evaluated inpatients.

In the present study, the same *S. capitis* clones colonizing community dwellers (nasal swabs) are probably also associated with hospital infections (blood cultures) and chronic wounds. These data confirm the high homogeneity of this species and the persistence of certain clones. A single clone that has been circulating from 2009 to 2012 might be associated with an outbreak of *S. capitis* involving patients with a positive blood culture and a wound infection. *Staphylococcus capitis* is not a common nosocomial pathogen and is rarely found in the microbiota of tested patients [43]. Nevertheless, this species is endemic in some neonatal units and has been associated with bloodstream infections [44].

### 3.8. S. pasteuri

Although the first description of this species is relatively recent, some studies have shown a high frequency among human and animal isolates [45,46]. *Staphylococcus pasteuri* is often misidentified as other species such as *S. warneri* [47]. Studies using sequencing of the 16S rRNA or *rpoB* gene report the presence of this species [45,46,47], while studies using biochemical tests or DNA amplification protocols may underestimate the presence of these isolates. In the present study, three unidentified strains were confirmed as *S. pasteuri* by *rpoB* gene sequencing. Two of the *S. pasteuri* nasal isolates were clonally related, probably because of the low prevalence of this species. One isolate carried SCC*mec* type I, as reported previously [48]. However, further analysis is not possible due to the low number of isolates in the present study.

### 3.9. S. simulans

Previous studies have shown high clonality of *S. simulans* [22,49], mostly associated with antimicrobial resistance, and some report specific heterogeneity [45]. The small number of *S. simulans* in the present study limits conclusions regarding the phylogenetic relationship among isolates of this species.

### 3.10. SCCmec

Proposing a criterion for clone definition based on PFGE, SCC*mec* typing, and MLST, Miragaia et al. [14] suggested that the best approach is to use PFGE typing followed by SCC*mec* determination. In fact, in that study, isolates belonging to the same PFGE type carried distinct SCC*mec* types. Such differences were also found in our study and might be related to the use of two typing techniques. Clearly, the high rates of recombination and frequent acquisition of mobile genetic elements favor the occurrence of genomic polymorphisms [14] not only in *S. epidermidis*, but also in other CoNS isolates.

SCC*mec* could not be typed in a considerable part of the isolates due to the amplification of two or more types of *ccr*/*mec* complexes, absence of amplification of one of the complexes, or combination of *mec*/*ccr* complexes that do not correspond to previously described types. Salgueiro et al. [26] observed 83% of *S. epidermidis* with non-typable SCC*mec*. The isolates with undetectable *ccr* complex type 2 and *mec* were positively associated with strains from bloodstream infections. In the present study, a considerable number of colonizing strains exhibited this SCC*mec* profile. However, since the PCR detection method for the two complexes was used only in the isolates that were not typed by the protocol of Machado et al. [33], we cannot make such a correlation for our isolates.

It is possible that the high recombination rates and the large number of element acquisition in CoNS [12,50], associated with high carriage rates of SCC*mec* [51], increase the potential for recombination into the cassette. In fact, analysis of the complete sequence of SCC*mec* by whole-genome sequencing of these strains with cassettes that are not typable by traditional amplification techniques would provide important data for the study of novel SCC*mec* types and recombination events.

In the study of Miragaia et al. [14], at least 18% of *S. epidermidis* isolates carried new SCC*mec* variants (non-typable elements or novel combinations of *mec*/*ccr* complexes), while other studies reported even higher frequencies [16,24]. In the present study, only one *S. epidermidis* isolate had a non-typed SCC*mec*, while most *S. hominis* and *S. warneri* isolates were not typable. This observation contrasts with descriptions for *S. aureus* in which few SCC*mec* types were identified in a large collection of isolates. The presence of similar regions in different SCC*mec* types indicates that the cassette has undergone several sequential events of recombination, giving rise to some structures resembling mosaics. It has been repeatedly suggested that a reservoir of SCC*mec* variants is being produced in CoNS and then transferred to *S. aureus* and other species [12].

Oliveira et al. [52] have also identified clones with 100% similarity that carried distinct SCC*mec* types, or clones with one resistant and one susceptible isolate. Interestingly, isolates belonging to the same PFGE type can carry up to three different combinations of the *mec* and *ccr* complexes and more than one SCC*mec* type, and several isolates can carry an SCC*mec* with multiple *ccr* complexes. A large proportion of isolates can carry residual SCC*mec*. These observations indicate that the acquisition and loss of mobile genetic elements in *S. epidermidis* are probably very common, even in the community environment [13].

The high diversity of SCC*mec* in CoNS might be related to frequent events of genetic transference, such as packaging and transduction by a bacteriophage and conjugation events. Some studies have observed transduction of SCC*mec* and other elements between *S. aureus* strains [53]. Bacteriophage transduction was considered the main mechanism of horizontal gene transfer between staphylococci, given the scarcity of mobilization and conjugation loci in staphylococcal plasmids [54]. In the last decade, new mechanisms of transference of plasmids were identified and might play an important role in genetic transference of mobile elements such as SCC*mec.* They include conjugation mediated by integrative and conjugative elements (ICEs), also known as conjugative transposons [55], and in trans recognition of multiple variants of the canonic origin of transfer (*oriT*) by some conjugative plasmids [56].

The present results regarding SCC*mec* diversity in CoNS may be biased since two methodologies were used. The high frequency of non-typable elements in CoNS may indicate that the elements of these species are so different from those of *S. aureus* that these typing methods should not be applied to these microorganisms [33,57].

## 4. Materials and Methods

### 4.1. Isolates

A total of 299 strains were studied. Of these, 132 were isolated from blood cultures obtained from inpatients admitted to the University Hospital of the Botucatu Medical School, UNESP, from 1990 to 2012, and were stored in the Culture Collection of the Department of Chemical and Biological Sciences, Microbiology and Immunology Sector, Biosciences Institute, UNESP. In addition, 145 strains were isolated from nasal swabs of healthy individuals from the same community in Botucatu (Sao Paulo, Brazil), and 22 were isolated from chronic wounds of patients attending basic health units in Botucatu (Sao Paulo, Brazil) from 2011 to 2013. The swab and wound isolates were obtained during previous projects of our group and were stored in the bacteriology laboratory at −80 °C. Sampling was designed in order to obtain the highest number of isolates from diverse staphylococcal species.

### 4.2. Identification of CoNS

The genus *Staphylococcus* was identified according to Baker [58] and Koneman et al. [59]. CoNS species were first identified by the simplified method proposed by Cunha et al. [60]. After DNA extraction with the Illustra kit (GE Healthcare) according to manufacturer instructions, the identification of strains was confirmed by the ITS-PCR technique using primers that target adjacent conserved sequences of the 16S rRNA and 23S rRNA genes [61]. The following reference strains were used for comparison of the results: *S. epidermidis* (ATCC 12228), *S. haemolyticus* (ATCC 29970), *S. capitis* subsp. *capitis* (ATCC 27843), *S. capitis* subsp. *ureolyticus* (ATCC 49325), *S. warneri* (ATCC 10209), *S. hominis* (ATCC 27844), *S. hominis* subsp. *novobiosepticus* (ATCC 700237), *S. lugdunensis* (ATCC 700328), *S. saprophyticus* (ATCC 15305), *S. schleiferi* subsp. *schleiferi* (ATCC 43808), *S. sciuri* subsp. *sciuri* (ATCC 29062), *S. simulans* (ATCC 27851), *S. xylosus* (ATCC 29979), and *S. caprae* (ATCC 35538). The standard strains were purchased from the American Type Culture Collection (ATCC, Manassas, VA, USA). The isolates showing discrepancy in the ITS-PCR band patterns were subjected to sequencing of the *rpoB* gene (nucleotides 1444–1928) [62].

### 4.3. PFGE Typing

PFGE typing was performed using a protocol modified from McDougal et al. [63]. The isolates were inoculated in BHI broth and incubated for 24 h at 37 °C. A 0.25 mL aliquot was centrifuged at 12,000 rpm for 50 s. The supernatant was discarded and 150 μL TE buffer (10 mM Tris, 1 mM EDTA (pH 8.0)) was added. This mixture was left in a water bath for 10 min at 37 °C. After vortexing, 2.5 μL lysostaphin (1 mg/mL in 20 mM sodium acetate (pH 4.5)) and 150 μL low melt agarose were added. The samples were added to plug molds, which were allowed to solidify. The plugs were transferred to 2 mL EC buffer (6 mM Tris-HCl, 1 M NaCl, 100 mM EDTA, 0.5% Brij-58, 0.2% sodium deoxycholate, 0.5% laurylsarcosyl sodium) and incubated at 37 °C for at least 4 h. The EC buffer was removed, and the plugs were washed four times in 2 mL TE for 30 min at room temperature. One-quarter of the plug was digested with *Sma*I in 50 μL of restriction buffer. The plugs were transferred to 1% agarose gels prepared in 0.5× TBE and covered with low melt agarose. Electrophoresis was performed at 6 V/cm for 21 h at 14 °C. Running conditions were initial switch time 5 s, final switch time 40 s, running time 21 h, voltage 6 V/cm. The comparison between gel patterns was made using UPGMA/Dice, tolerance 1.25%, optimization 1%, and similarity 80%. The data were analyzed with the Bionumerics 7.1 software (Applied Maths, Sint-Martens-Latem, Belgium).

Since the *S. hominis* isolates were not properly restricted with *Sma*I, revealing patterns with fewer than 5 bands, the protocol of Bouchami et al. [32] based on restriction with *Xho*I was used. Running conditions were the following: block1—pulse times 2 to 20 s, running time 11 h; block2—pulse times 2 to 7 s, running time 15 h; voltage 6 V; angle 120°. For *S. hominis*, UPGMA/Dice was also used, with 1% of tolerance and 0.8% optimization, using a cutoff similarity value of 80%. The data were analyzed with the Bionumerics 7.1 software.

### 4.4. SCCmec Typing

SCC*mec* typing was performed by multiplex PCR using the primers described by Machado et al. [33] (Protocol 1). For isolates not typed by Protocol 1, the multiplex PCRs 1 and 2 described by Kondo et al. [64] (Protocol 2) were used. The SCC*mec* was defined as non-typable when (a) Protocol 1 showed no amplification product and (b) in Protocol 2 (b1) the *ccr* complex exhibited no amplification product other than the *mecA* gene (286 bp), or (b2) the *mec* complex showed no amplification band, (b3) or the combination of *ccr/mec* complexes determined an unknown SCC*mec* type. In the case of amplification of more than one fragment by Protocol 1, the isolate was considered to carry two SCC*mec* types.

### 4.5. MLST

MLST of *S. epidermidis* was performed according to Thomas et al. [65]. The following seven housekeeping genes were used: carbamate kinase (*arcC*), shikimate dehydrogenase (*aroE*), ABC transporter (*gtr*), DNA mismatch repair protein (*mutS*), pyrimidine operon regulatory protein (*pyrR*), triosephosphate isomerase (*tpiA*), and acetyl coenzyme A acetyltransferase (*yqiL*). The PCR products were purified, precipitated, and sequenced in an ABI Prism 377 sequencer (Applied Biosystems). The Bionumerics Sequence Typing and MEGA 6.0 software were used to align the nucleotide sequences. The sequences were compared by database searches via the http://www.mlst.net website, accessed on 2 May 2021. The *S. epidermidis* sequence types were deposited in the MLST database (pubmlst.org, accessed on 2 May 2021).

### 4.6. Diversity Index

The Simpson diversity index was calculated as the number of strains per clonal profile according to Hunter and Gaston [66]. This index is defined as the probability of two unrelated strains originating from a given population being assigned to different groups.

## 5. Conclusions

The present study demonstrated the presence of CoNS clones circulating among hospital and community environments, especially *S. lugdunensis* and *S. warneri* isolates. Some PFGE types, such as *S. haemolyticus*, *S. epidermidis*, and *S. capitis* isolates, were restricted to one of the environments. The high diversity of SCC*mec* types within a clone suggests that SCC*mec* may not be a marker of the phylogenetic diversity of CoNS. The most prevalent species, *S. epidermidis*, exhibited STs associated with antimicrobial resistance and novel types [57].

## Figures and Tables

**Table 1:** SCC*mec* types according to sampling site.

SCC*mec*	Nasal Colonization	Wounds	Blood Cultures
I	25 (52)	9 (42.8)	32 (27.6)
II	1 (2)	0	1 (0.9)
III	3 (6.25)	0	9 (7.8)
IV	1 (2)	0	9 (7.8)
V	3 (6.25)	0	12 (10.3)
I, III	4 (8.3)	0	16 (13.8)
I, II	3 (6.25)	4 (19)	6 (5.2)
III, IV	0	0	9 (7.8)
VIII	1 (2)	0	0
NT	7 (14.6)	8 (38)	21 (18.1)
*mecA* negative	97 (67)	1 (4.5)	16 (12.1)
Total	145 (100)	22 (100)	132 (100)

NT = not typed.

**Table 2:** Amplification products detected in *mecA*-positive isolates without the *mec* or *ccr* complex or complex multiplicity.

Isolate	Species	Protocol 2		
		IC	*ccr*	*mec*
78A	*S. epidermidis*	+	1 + 4	A
83C	*S. hominis*	+	1	A
93C	*S. hominis*	+	1	A
116C	*S. warneri*	+	1 + 4 + 5	−
139C	*S. capitis*	+	−	A + B

IC = internal control for *mecA*. + = positive, − = negative.

**Table 3:** Diversity index of the CoNS species.

Species	Diversity Index
*S. epidermidis*	0.971026
*S. haemolyticus*	0.984708
*S. warneri*	0.981087
*S. hominis*	0.995717
*S. capitis*	0.969416
*S. lugdunensis*	0.977062
*S. saprophyticus*	0.999598
*S. pasteuri*	0.999598

## Data Availability

The data presented in this study are contained within the article.
